# Current Status of Direct Acting Antiviral Agents against Hepatitis C Virus Infection in Pakistan

**DOI:** 10.3390/medicina54050080

**Published:** 2018-11-05

**Authors:** Saba Khaliq, Syed Mohsin Raza

**Affiliations:** 1 Department of Physiology and Cell Biology, University of Health Sciences, Lahore 54600, Pakistan; 2 Institute of Biomedical and Allied Health Sciences, University of Health Sciences, Lahore 54600, Pakistan; smraza@uhs.edu.pk

**Keywords:** direct-acting antivirals agents, sustained virological response (SVR), interferon-free, resistance, hepatitis C virus

## Abstract

In Pakistan, the burden of the hepatitis C virus (HCV) infection is the second highest in the world with the development of chronic hepatitis. Interferon-based combination therapy with ribavirin was the only available treatment until a few years back, with severe side-effects and high failure rates against different genotypes of HCV. Interferon-free all-oral direct-acting antiviral agents (DAAs) approved by the FDA have revolutionized the HCV therapeutic landscape due to their efficiency in targeting different genotypes in different categories of patients, including treatment naïve, treatment failure and relapsing patients, as well as patients with compensated and decompensated cirrhosis. The availability and use of these DAAs is limited in the developing world. Sofosbuvir (SOF), a uridine nucleotide analogue and inhibitor of HCV encoded NS5B polymerase, is now a widely available and in-use DAA in Pakistan; whereas daclatasvir was recently added in the list. According to the documented results, there is hope that this disease can be effectively cured in Pakistan, although a few concerns still remain. The aim of this article is to review the effectiveness of DAAs and the current status of this treatment against HCV genotype 3 infection in Pakistan; various factors associated with SVR; its limitations as an effective treatment regime; and future implications.

## 1. Introduction

Since its discovery in 1989, chronic hepatitis C virus (HCV) infections have become the most common global health problem, with current reports stating more than 71 million infected people worldwide [1]. As infected individuals with this disease are often asymptomatic, more than 50–80% of the patients are unaware of their disease. Chronic hepatitis C (CHC) may lead to cirrhosis, decompensated liver disease, and hepatocellular carcinoma (HCC) over a period of 20–30 years in 30–50% infected patients, with approximately 3.5–5 million people estimated to die each year due to these complications [2]. The prevalence of HCV varies worldwide, with the highest incidence in the Middle East and North African regions, including Egypt and Pakistan [3]. Pakistan has the second-largest HCV burden in the world, with recent estimates suggesting that Pakistan has an estimated adult HCV sero-prevalence of 4.5–8.2% [4,5]. Out of the six major HCV genotypes, the majority of HCV infections in Pakistan are genotype 3a (69.1%), followed by genotypes 1 (7.1%), 2 (4.2%), and 4 (2.2%) [5]. In Pakistan, HCV transmission is mainly driven by multiple risk factors, such as health care practices (blood transfusion and injections with a prevalence of 27–42.3% in health care professionals and 7.8–68% in the general population), community-based activities (barbering, ear/nose piercing), and injecting drug use [5,6,7].

Conventional interferon (IFN) monotherapy has been used since 1991, which was followed by pegylated-interferon (PEG-IFN) plus ribavirin (RBV) treatment in 2001. Using these treatments, only 40–45% sustained virological response (SVR) was achieved against genotype 1, up to 80% against genotype 2, and only 50% against genotype 3a. Collectively, this therapy resulted in about 50–60% of HCV infected patients not responding or relapsing [8]. Despite the extensive Chief Minister’s Program for Hepatitis B and C Control in Pakistan where the IFN-based treatment was used, viral clearance has been documented in only 67% of the infected population [9]. Moreover, this treatment regime was associated with troublesome side effects such as flu, fatigue, fever, and leucopenia or thrombocytopenia, leading to dose reduction or discontinuation of treatment [10,11]. Limited efficacy and the associated side effects encouraged the development of new therapeutic regimes with better SVR rates and minimal side effects. 

During the past decade, the treatment options against hepatitis C have dramatically increased due to the development of IFN-free oral treatment regimen that directly acts on the HCV non-structural proteins, called direct-acting antiviral agents (DAAs). These DAAs have revolutionized the management of hepatitis C, as they are well-tolerated and achieve cure rates of over 90% regardless of liver fibrosis, prior response to IFN/RBV, gender, age, and race [12]. Sofosbuvir (SOF), the first nucleotide analogue, is also effective without IFN treatment [13]. HCV genotype 3 is emerging as being difficult to treat, especially for those who have had previous treatment or have developed cirrhosis. Very few drugs are effective for the treatment of these types of patients, with SOF being one of them [14,15]. Different studies have reported that 12 weeks treatment of SOF + RBV with and without PEG-IFN resulted in a substantial decrease in the viral RNA, leading to a SVR in 92% against HCV genotype 2 or 3 infected patients after 24 weeks of treatment [16,17,18]. The data showed that the treatment response of oral regimens is promising with some concerns in the Pakistani population, where the predominant genotype is 3a.

## 2. Literature Review Strategy

PubMed and Google Scholar were searched using the MeSH terms “Hepatitis C, Chronic/drug therapy”, “Direct-acting antivirals”, “Resistance to DAAs”, “HCV and Pakistan”, “Direct-acting antivirals and Pakistan”, and “Treatment of HCV in Pakistan”. The search was filtered to only include English language articles. Articles, including original articles, abstracts, conference proceedings, and review articles, were screened. Studies within the past 5 years focusing on DAAs were mainly selected. Important older publications with common references were included, but articles focusing only on interferon therapy were not considered. The latest guidelines of the American Association for the Study of Liver Disease (AASLD, 2016) and the European Association for the Study of Liver (EASL, 2017) were also reviewed for updating the existing information on the topic.

## 3. DAAs against All Genotypes

The basic research on the HCV structure and replicative cycle has revolutionized the development of DAAs, resulting in an increase in the SVR rates from 40–50% to more than 95%. The first-generation DAA targeting NS3/4APIs (boceprevir and telaprevir) was approved for clinical use in 2011. After the achievement of positive results, this new standard treatment became more efficient due to the development of a triple therapy consisting of PEG-IFN/RBV and either boceprevir (BOC) or telaprevir (TVP). This achieved a SVR in 60–70% of the patients with HCV genotype 1 [19]. The treatment of HCV has undergone revolutionary changes, and the FDA has approved the following second-generation DAAs to be used in clinical practice against different genotypes:

- Simeprevir (NS3/NS4A protease inhibitor): approved for genotype 1 and 4 [20,21,22];
- Sofosbuvir (NS5B inhibitor): approved for genotypes 1, 2, 3, and 4 in treatment-naïve, treatment-experienced, compensated cirrhosis, and in patients with HCC [18,23,24,25];
- Daclatasvir (NS5A inhibitor): approved for genotypes 1, 2, 3, and 4 [26,27];
- Glacaprevir with pibrentasvir (NS3/NS4A protease and NS5A inhibitor): approved for all genotypes [28,29,30,31];
- Sofosbuvir and daclatasvir (NS5B and NS5A inhibitor): approved for genotypes 1, 2, 3, and 4 [32,33,34];
- Sofosbuvir with velpatasvir (NS5B and NS5A inhibitor): approved for all genotypes with compensated and decompensated liver [35,36,37,38,39,40,41,42,43,44];
- Sofosbuvir with ledipasvir (NS5B and NS5A inhibitor): approved for genotype 1 with renal impairment [45,46,47];
- Elbasvir with grazoprevir (NS5A and NS3/NS4A protease inhibitor): approved for all genotypes and patients with severe renal impairment [48,49,50,51,52];
- Ombitasvir with paritaprevir and ritonavir (NS5A, NS3/NS4A inhibitor and HIV antiretroviral drug): approved for genotype 1 and 4 patients coinfected with HIV [53,54];
- Paritaprevir, ombitasvir, and dasabuvir with ritonavir (NS3/NS4A, NS5A, NS5B inhibitor with HIV antiretroviral drug): approved for genotype 1 patients coinfected with HIV [55].

The introduction of SOF in the treatment regime has achieved groundbreaking success [56]. In liver, the phosphorylated form of SOF targets the highly conserved active site of the NS5B polymerase, thereby causing the termination of the RNA replication of the virus [57,58]. It has improved the SVR rate in different genotypes, which is 100% for genotype 2 and up to 65–80% in genotype 3 for both treatment-naïve and treatment-experienced patients [25,59,60].

## 4. Availability of DAAs against HCV in Pakistan

In Pakistan, the first-line treatment for chronic HCV infections is changing to the new DAAs. This includes SOF + RBV, which has been one of the registered and widely available DAAs in Pakistan since November 2014. SOF has also been associated with fewer side effects compared to IFN-based therapy [61,62]. Despite being effective, SOF use is limited due to its high price in Pakistan—a country where the majority of the HCV infected population is in the lower income category. The government of Pakistan and the pharmaceutical companies manufacturing SOF have agreed to provide it at a substantially reduced price, as Pakistan is amongst the high-burden countries for HCV [63]. Moreover, the inclusion of an SOF-based treatment regimen in the National Guidelines for HCV Treatment in Pakistan has increased its use by clinicians. At the same time, the use of DAAs has not been limited to the SOF-based treatment regimen and new more effective DAAs have been welcomed in Pakistan. Recently, daclatasvir (DCV) has also been made available in Pakistan and incorporated in the treatment regimen. Such additions will definitely offer better safety profile with improving patient adherence [64].

## 5. Preliminary Data Regarding the Success Rate of DAAs against HCV Genotype 3

The data regarding SVR in HCV genotype 3 are variable in different populations and different pathological conditions. In treatment-naïve patients with genotype 3, the SVR12 (SVR after 12 weeks of treatment) rate was only 56% [25]. The ELECTRON study focusing on the use of SOF/RBV in genotype 2 and 3 patients without cirrhosis showed 100% SVR24 (SVR after 24 weeks of treatment) [62]. In another study, the SVR24 was 61% for genotype 3 and 93% for genotype 2 in non-cirrhotic patients, respectively [23]. A retrospective study reported SVR rates of 81% in genotype 3 patients after 24 weeks of treatment [65]. However, two recent studies on the Indian population with genotype 3 reported SVR12 rates of 96–98% regardless of the severity of the disease and whether the patients were treatment naïve or treatment experienced [66,67]. Overall, these reports encouraged the addition of SOF in the treatment regime to eliminate the hepatitis C disease from the Pakistani population, which predominantly is of genotype 3 [5,68].

Different studies from Pakistan, which were mainly from the Punjab province, reported the efficacy of SOF-based therapies in HCV genotype 3-infected patients (Table 1). SOF-based dual or triple therapy has so far shown to be very effective in genotype 3 patients, with a SVR24 of 82.2–99.34%. However, the results are suboptimal, especially in patients with a decompensated liver and with or without significant fibrosis [69,70,71,72]. So far, the largest study with a cohort of 1375 patients from Lahore performed during 2014 to 2016 has also shown a remarkable SVR rate of 97–99% in genotype 3-infected patients after SOF treatment as double or triple therapy regime [73].

Hemodialysis is a risk factor for the HCV infection, as the prevalence of HCV is 16.4–68.0% in Pakistani hemodialysis patients [74]. In a local study, SOF was used along with RBV for 24 weeks and a 100% SVR rate was observed [75]. This is the first study to report the efficacy of SOF + RBV combination therapy in 37 treatment-naïve and treatment-experienced hemodialysis patients. Sofosbuvir and ribavirin combination therapy not only efficiently eradicated HCV genotypes 1, 2, 3, and 4, but was effective in renal transplant recipients (RTRs) which had mixed infection with HCV genotypes 1 and 3 [75].

During DAAs treatment, continuous monitoring of viral load is recommended. Saleem et al. (2018) reported five cases that showed unusual response toward the use of DAA with abruptly high viral titers and liver function tests (LFTs) in patients at 12 weeks post treatment. Although 6-month DAA combination therapy with ribavirin cleared HCV, the patients experienced an abrupt and marked rise in viral loads during the initial months of treatment followed by the sudden elimination of virus during last 2 months of treatment [76].

Although the overall successful SVR rates observed in the Pakistani population encourage the use of SOF in HCV-infected patients, there are still some exceptions leading to HCV relapse and non-responsiveness toward DAAs which should be considered before the use of DAAs in our population. A single-center study conducted on 100 patients, who relapsed after 24 weeks of treatment with SOF + RBV therapy, reported that addition of daclatsvir to the treatment regime (SOF + RBV therapy) resulted in 94% SVR in these patients after 24 weeks [78]. Wahid et al. (2018) [77] reported a case report of a 77-year-old female patient diagnosed and treated with IFN-based therapy against HCV genotype 3a in January 2011. There was a relapse of disease in 2015 and she was treated with SOF + RBV therapy. A decrease in viral load was observed during the first 3 months of treatment, but after 3 months of treatment the quantity of serum HCV RNA started increasing until the end of treatment (24 weeks). The patient was then screened for HBV and HIV, and it was later on confirmed that she was not co-infected with either HIV or HBV but for *Mycobacterium tuberculosis* (MTB). The patient was then referred to a pulmonologist, and after successful anti-tuberculosis (TB) treatment, anti-HCV therapy based on different interferon-free regimen (i.e., sofosbuvir and daclatasvir) was prescribed that led to the elimination of virus in 12 weeks. Wahid et al. (2018) further elaborated the case, as the continuous destruction of immune cells and active immune suppression caused by MTB hinder HCV treatment with DAA drugs. Therefore, in order to successfully eliminate HCV in HCV–MTB co-infected patients, healthcare professionals need to treat TB before treating HCV. Sofosbuvir, daclatasvir, and ribavirin is the effective treatment regimen in such patients [77].

## 6. Concerns to Be Addressed before the Administration of DAAs

Although the initial reports of the use of SOF against HCV genotype 3 in the Pakistani population are very promising in eliminating this deadly infection, there are a few concerns, especially resistance against the antiviral treatment, which should be taken into account before the start of therapy.

### 6.1. Hepatitis C Viral Resistance to DAAs

The HCV-infected individual has a mixture of genetically similar strains of HCV, with a predominant drug-sensitive wild-type strain and a low number of resistant strains. These drug-resistant strains are due to a mutant amino acid in the viral genome, which enhances virus survival and renders DAA less effective against these strains [79]. Most well-documented resistance-associated strains against SOF show L159F, V321A, and S282R variations [80]. Thus, DAA resistance is a rising issue warranting further evaluation in both treatment-naïve and treatment-experienced patients. Pre-treatment resistant studies are a part of the recommendations given by AASLD 2016 guidelines to evaluate NS5A-resistant variants in patients that failed DAA treatment, especially for elbasvir/grazoprevir against genotype 1a patients [81]. Aziz et al. (2018) [82] reported the presence of no S282T variant in NS5B region of HCV genotype 3 and 1 in patients with and without response to SOF + RBV-based 24-week therapy in a Pakistani population. Although most of the studies have shown a success rate of up to 99%, more in-depth studies are required in order to conduct the detailed analysis of patients with failure of DAA treatment and their follow-up studies in the case of a relapse, as the resistant strain may prevent patients from achieving SVR and any patient with DAA failure should be managed according to the latest recommended guidelines [83].

### 6.2. Host Genetic Variation and DAAs

Recently, Zia et al. [84] reported a case study describing a 47-year-old male patient who was treated twice with traditional IFN + RBV therapy in 2007 and 2010, followed by two additional cycles of PEG-IFN α-2b + RBV in 2011 and 2013. In 2016, the patient underwent SOF + RBV combination therapy and unfortunately, SVR was still not achieved. In terms of host genetic factor, this patient was found to be heterozygous rs8099917G/T and rs12979860C/T for the IL28B polymorphisms, respectively. Phylogenetic analysis suggested that the resistant variant belonged to an out-group and may require triple therapy. Zia et al. recommended a triple therapy (RBV + IFN + SOF/BOC or RBV + BOC + SOF) for this case, which may be effective against HCV in this particular case. There are a number of studies from Pakistan that reported the involvement of the IL28B genetic variant in the failure of IFN-based therapy [85,86,87,88], so more studies are needed to completely understand the involvement of multidrug-resistant HCV variants and host genetic factors in the Pakistani population [84].

### 6.3. Risk of HCC and DAAs

Recently, a controversy regarding the higher incidence/recurrence of HCC post-DAAs treatment has been highlighted in different studies. An unexpected high incidence of HCC recurrence in patients achieving SVR with DAAs has been reported in different retrospective studies [89,90,91,92,93]. Conti et al. (2016) [90] indicated different factors (i.e., patient characteristics and treatment duration/timing) which may be contributing to the HCC recurrence in DAAs-treated patients. HCC recurrence is reported to be related with the time interval between HCC treatment and antiviral therapy, with a greater incidence in the first month post-HCC treatment [94,95]. These reports are either case reports or are based on limited data, so extensive research is needed to prove any relationship of HCC with the use of DAAs. Physicians around the world should keep these possibilities in mind and promptly report any adverse events to the FDA [83]. Recently, the results of a retrospective case–control study showed no impact of DAAs on the recurrence of HCC, characteristics of HCC, and time interval [96]. Adhoute et al. (2018) [96] suggested that the time interval between cancer treatment and DAAs treatment may be due to undetected recurrence or disruption of immune surveillance, so HCC and DAAs treatments should be 12 months apart to possibly avoid the recurrence.

Furthermore, post-DAAs treatment, 29 cases were reported to the FDA from 2013 to 2016 showing the reactivation of hepatitis B infection in patients treated with DAAs having HBV–HCV co-infection [97]. The FDA thus boxed a warning to screen all patients for HBV co-infection before, during, and after treating HCV with DAAs [19]. The exact mechanism behind the reactivation of HBV in DAAs treated patients is still unclear, however it is suggested to be a result of HCV clearance from the body, lack of DAAs specificity against HBV, and immunological response [98].

### 6.4. Prime Problem in Eliminating HCV in Pakistan

Irrespective of all the therapeutic options, the major problem with all HCV elimination programs is the identification of infected individuals, which needs considerable screening of the general population. Moreover, the healthcare infrastructure in Pakistan and other low-income countries needs improvements in order to achieve proper diagnoses as well as prevention and control measures for reducing HCV-related mortality [99]. Another problem is the limited number of certified and trained hepatologists and gastroenterologists in the country. Keeping our local scenario in mind, the proper training of general practitioners should be arranged, and they should be encouraged to follow the guidelines set by the FDA and the Pakistan Medical and Dental Council (PMDC). Furthermore, there should be timely referrals for complications to specialized hepatologists and gastroenterologists. Although no adverse events have been reported that are related to the prescription of HCV-related DAA therapy by non-specialist practitioners, their proper training would prevent any such event in the future [83], because there is a great scarcity of therapeutic response data from Pakistan.

## 7. Future Perspectives

The use of SOF and RBV with or without IFN in CHC patients is efficacious and safe for both compensated and decompensated liver. SOF has a number of beneficial qualities, especially its pan-genotypic activity, fewer adverse effects, minimal drug–drug interactions, a high genetic barrier to resistance, efficacy in patients with advanced liver disease, and excellent SVR rates in treatment-naïve and treatment-experienced patients. Further studies are needed for understanding the associated host and viral factors, with a special focus on the difficult-to-treat viral variants (i.e., relapsing patients and non-responders) in the Pakistani population. Triple therapy of SOF + DCV + RBV is very promising in relapsers of SOF + RBV combination, but multicenter studies are needed to confirm this observation in our population. More trials and reports will improve the outcome of the disease and help to eradicate HCV. There are a few concerns (e.g., HCV-resistant variants against DAAs, host genetic profile, and co-infection) that should be considered before the start of therapy. Being a resource-limited country, it is strongly recommended to screen all the patients for co-infection (especially HBV, HIV, and TB) before the start of DAA-based therapy and selection of better/combination of different DAAs available in Pakistan. Several treatment regimens are in clinical developmental stages, which would increase SVR with minimal side-effects, improved tolerability, and a high barrier against viral resistance, and could be a part of patients’ management in the near future.

## Figures and Tables

**Table 1:** Summary of the results of sofosbuvir in Pakistani patients infected with HCV genotype 3. SVR12/24: Sustained virological response after 12 or 24 weeks of treatment; Hb: hemoglobin; LFT: liver function test; PEG-IFN: pegylated-interferon; RBV: ribavirin; SOF: sofosbuvir; ALP: Alkaline Phosphatase; ALT: Alanine Aminotransferase; AST: Aspartate Aminotransferase.

Study	Patient Population/Region (*n* = Sample Size)	Treatment Regime	SVR 12/24	Side-Effects/Disadvantage
Akhter et al., 2016 [70]	Treatment naïve/Rawalpindi (*n* = 198)	SOF + RBV	SVR 12 (89.2%)	Fatigue, generalized weakness, headache, and anemia related to ribavirin
Siddique et al., 2017 [72]	Treatment naïve and treatment experienced/Karachi (*n* = 201)	SOF + RBV SOF + RBV + PEG-IFN alfa-2a	SVR 24 (88.5%) SVR12 (98.5%)	No side effects reported
Sarwar et al., 2017 [71]	Treatment naïve and treatment experienced/Lahore (*n* = 216)	SOF + RBV SOF + RBV + PEG-IFN alfa-2a	SVR 24 (82.2%) SVR12 (89.2%)	Fatigue, headache and fever. Adverse effect was worsening of ascites (4.6% of patients)
Azam et al., 2017 [69]	Treatment naïve and treatment experienced/multi-center study (*n* = 573)	SOF + RBV SOF + RBV + PEG-IFN alfa-2a	Rapid virological response (RVR) (98.2%)	Fatigue and diarrhea, flu-like symptoms, and other adverse effects
Iqbal et al., 2017 [73]	Treatment experienced and treatment naïve/Lahore (*n* = 1375)	SOF + RBV SOF + RBV + PEG-IFN alfa-2a	SVR 24 (99.34%) SVR12 (93.46%)	Headache, fatigue and anemia Less effective in old age, Headache, fatigue and anemia, Myalgia, decreased appetite, hair loss, aggression, depression, leukocytopenia and thrombocytopenia
Hanif et al., 2017 [75]	Hemodialysis patients/Karachi (*n* = 37)	SOF + RBV	SVR24 (100%)	No side effects reported
Wahid et al., 2018 [77]	Treatment experienced/Lahore (*n* = 1, tuberculosis (TB) co-infected patient)	SOF + RBV SOF + daclatasvir	Non-responder SVR12	No side effects reported
Saleem et al., 2018 [76]	Treatment naïve/Lahore (*n* = 5)	SOF + RBV	SVR 24	Increased LFTs Mild change in ALP, ALT, AST, bilirubin, Hb, and platelets
